# Lower class competence stereotypes of the upper class increase class conflict: mediation by intergroup envy and moderation by upward social mobility belief

**DOI:** 10.3389/fpsyg.2024.1360951

**Published:** 2024-05-30

**Authors:** Jia-Ling Liu, Lei Yan, Yan-Hong Zhang, Jin-Hua Gan, Lin-Chuan Yang

**Affiliations:** ^1^College of Education and Sports Sciences, Yangtze University, Jingzhou, Hubei, China; ^2^Social Psychology Research Center of Yangtze University, Jingzhou, Hubei, China; ^3^College of Education, Three Gorges University, Yichang, China

**Keywords:** competence stereotypes, class conflict, intergroup envy, upward social mobility belief, social identity theory

## Abstract

**Background:**

With increasing gaps between the rich and poor, potential risk factors for class conflict have attracted increasing attention from researchers. Although cognitive factors are known to be significant predictors of class-conflict behavior, limited attention has been paid to competence stereotypes of the upper class. When considering economic inequality, people pay more attention to competence stereotypes of the upper class, which may have adverse effects. This study aimed to investigate the relationship between competence stereotypes held by the lower class about the upper class and class conflict, and to test the mediating role of intergroup envy in this relationship and the moderating role of upward social mobility belief.

**Methods:**

Data were collected from a convenience sample from a comprehensive university in China. Based on scores on subjective and objective class scales, 284 lower-class college students (103 males and 181 females) aged 18–24 were selected to participate (both their subjective and objective scores were lower than 3 points). Their endorsement of upper-class competence stereotypes, intergroup envy, upward social mobility beliefs, and class conflict were measured using a well-validated self-report questionnaire.

**Results:**

The main data were analyzed using correlation analysis, the SPSS macro PROCESS (Model 7), and simple slope analysis. The results show a significant positive correlation between competence stereotypes held by lower-class college students toward the higher class and class conflict, and this connection was mediated by intergroup envy. Moreover, the indirect effect of intergroup envy on this link was moderated by upward social mobility beliefs; this effect was stronger for college students with lower upward social mobility beliefs.

**Conclusion:**

This study broadens our understanding of how and when competence stereotypes among the lower class concerning the upper class are related to class conflict. Researchers and policymakers should pay special attention to competence stereotypes of the upper class, especially intergroup envy and class conflict among lower-class individuals with lower levels of upward social mobility beliefs.

## 1 Introduction

Economic development is accompanied by social change. Individuals classify themselves into social classes based on objective material resources and subjective social status differences (Hu et al., 2014). In recent years, the intensification of economic inequality has made the differentiation of upper and lower social classes in countries around the world increasingly apparent (Brian, 2015). The gap between rich and poor in upper and lower social classes is increasing, causing the wealth of a few upper-class people to be dozens of times that of the lower class (Coffey et al., 2020). This extreme degree of inequality poses a threat to society at the group level (Tanjitpiyanond et al., 2022). Studies have shown that severe inequality is associated with a series of negative social consequences (e.g., reduced social trust and increased class conflict) (Kraus et al., 2017; Jetten and Peters, 2019; Gordils et al., 2020; McGovern et al., 2021). Class conflict refers to group behavior caused by perceived differences in interests among different classes (Pruitt and Sung, 2013; Dahrendorf, 2019). Such interests include material interests (such as money), social interests (such as power), and psychological needs (such as respect) (Pruitt and Sung, 2013). People who engage in class conflict behaviors often belong to lower-class groups. For example, in group events, the lower class participates in rights protection events and protests, as well as strikes or demonstrations against employers, capitalists, governments, etc. (Van Zomeren et al., 2008; Piven and Cloward, 2012; Lindström et al., 2023). There are two reasons for this collective behavior in lower-class groups. One is objective dissatisfaction with their material needs, and the other is the lack of fulfillment of subjective psychological needs (Sainz et al., 2021). Studies have shown that, as social competition has become fiercer, awareness of class conflict among lower-class groups has gradually increased (Zhang and Liu, 2017). Membership of lower-class groups is much larger than that of upper-class groups (Sicular et al., 2022). Under unequal economic development, large-scale class conflicts occur readily (Bircan et al., 2017). To promote the harmonious development of society as a whole, it is particularly important to study the internal mechanisms of class conflict caused by lower-class groups. Therefore, it is of great value to explore the potential psychological factors and internal mechanisms of conflict in lower class people.

A group’s social cognition can affect individual psychology and behavior (Hu et al., 2014). In recent years, the rise in economic inequality has increased the prominence of class categories based on wealth (Peters and Jetten, 2023). Frequent group comparisons between different classes enhance the perception of in-group versus out-group (Jetten et al., 2021). As a potential threat factor affecting intergroup cooperation and conflict, class stereotypes inherent in all classes have gradually been paid attention by researchers (Borinca et al., 2021; Tanjitpiyanond et al., 2022). Class stereotypes are fixed mental schemas about different classes based on class categorization (Liu and Zuo, 2013). Class stereotypes play an important role in class interactions and can broadly affect people’s cognition, emotions, and behavior (Cuddy et al., 2007). Research on class stereotypes shows that lower-class groups are often characterized as lazy and drug abusers, resembling animals (Loughnan et al., 2014; Lindqvist et al., 2017), whereas upper-class groups are characterized as more capable, smarter, and more self-disciplined (Wu et al., 2018; Connor et al., 2021). These specific stereotype traits are summarized by Fiske’s Stereotype Content Model (SCM) into two dimensions: “warmth” and “competence.” Warmth refers to “kindness” in the intentions underlying group behavior, which depends on perceived willingness to cooperate or the threat of competition. “Competence” indicates the capability of a group to realize its intentions, which is dependent on the relative position within its social class (e.g., income, wealth, education, or work prestige) (Fiske et al., 2002). Research on class stereotypes has shown consistent results in many countries; based on SCM, lower-class stereotypes are characterized by high warmth–low competence, and upper-class stereotypes are characterized by low warmth–high competence (Tanjitpiyanond et al., 2023). However, Wu et al.’s (2018) study of wealthy class stereotypes found that people have various stereotypes of different upper-class groups. Specifically, people’s stereotypes of engineers and CEOs are high competence and high warmth, whereas the stereotype of the rich second-generation and government officials is high competence and low warmth. The study also found that different stereotypes of these two higher class groups triggered different emotional and behavioral responses. The current study focuses on how low-warmth–high-competence stereotypes held by the lower class toward the higher class can generate social emotions and behaviors.

Different class stereotypes can cause different social emotional and behavioral reactions (Cuddy et al., 2007; Chen et al., 2023). According to the Behaviors from Intergroup Affect and Stereotypes(BIAS) Map theory, to promote their survival or interests, people instinctively confirm whether other groups are enemies (warmth) and whether they can pose a threat (competence) based on the degree of the competition and relative status between groups, thus forming stereotypes of different groups. Research shows that the public’s stereotype of high-class groups (such as the rich, urban people) is “low enthusiasm–high competence,” which often evokes jealousy and envy, and triggers the behavioral orientation of cooperation and connection (Fiske et al., 2002; Cuddy et al., 2007). However, as members of the public, lower-class groups often engage in aggressive behavior in mass incidents (e.g., rights protection events and protests) (Osborne et al., 2022; Lindström et al., 2023). Confronted with inconsistent results, some researchers try to explain the two dimensions of stereotypes. It is proposed that the aggressive behavior of the lower classes is caused by the low warmth stereotype of the upper classes (Sevillano and Fiske, 2019). The BIAS map theory underscores warmth as the primary dimension that significantly influences emotions and behaviors, eliciting “active facilitation” behavior, whereas its absence can trigger “active harm” behavior. Conversely, competence serves as a secondary dimension, inducing “passive facilitation” behavior and, in its absence, leading to “passive harm” behavior (Cuddy et al., 2007). However, few studies have explored this explanation further.

Based on many studies, we find that in the context of economic inequality, people’s perceptions of class competence stereotypes have become stronger (Connor et al., 2021). Tanjitpiyanond et al.’s (2022) study manipulated people’s perceptions of economic inequality, and found that individuals who perceived high economic inequality cared more about a group’s competence stereotype. This may indicate that an individual’s competence stereotypes can better predict behavioral responses. Canton et al. (2023) investigated the competence stereotypes and emotional and behavioral responses of people with disabilities. The public holds the low competence stereotype of the disabled person, which will produce the pity, the sympathy emotion, and the active help behavior response. Unkelbach et al. (2023) study found that lower social class of individuals, for political officials on the face of perceived ability, will affect their support for political officials. Therefore, in the context of economic inequality, this study directly explores whether the competence stereotypes held by specific groups (such as lower-class groups) toward other groups (such as upper-class groups) can lead to harmful behaviors.

The competence stereotypes of the higher class may be an important cause of class conflict. Firstly, in the face of increasing competition for scarce resources, lower class groups are more likely to cause class conflict. According to the theory of realistic conflict, in the face of competition for scarce resources (e.g., power, status, and prestige), groups are prone to populationism and hostility (Tajfel and Turner, 2004). In special situations, lower-class groups exhibit more aggressive behavior (Chen, 2022). Against the backdrop of economic inequality, McGovern et al. (2021) conducted separate investigations among White and Black Americans, focusing on individuals with a significant objective income gap. Their findings revealed that Black Americans exhibited a lower level of interracial trust and predicted a higher level of social conflict. Zhang (2013) found that lower class people exhibited a higher cluster behavioral intention in a virtual situation of land distribution. Cluster behavioral intention refers to the dissatisfaction of group members with the current status of the group, leading them to engage in actions aimed at improving the group’s status quo (Wright et al., 1990; Wright, 2009). Therefore, it is speculated that when individuals with low social status are unable to obtain scarce resources, they will strengthen their hostility toward the upper class, leading to class conflict.

Second, lower-class groups experienced more real threats. In the case of unequal economic development, lower class groups not only experience more difficulties in obtaining power, economic resources, and so on, but also experience a sense of threat from upper-class groups to their ability to obtain resources (Kraus et al., 2013). When a lower-class group experiences real threats, it demonstrates more hostile behavior (Giani and Merlino, 2021). Makashvili et al. (2018) found that compared with individuals who did not experience real threats, individuals who experienced real threats showed higher negative prejudice toward external groups. Negative prejudice tends to predict aggressive behavior (Sevillano and Fiske, 2019). A study by Martinez et al. (2022) of manipulation threat types and aggressive behavior also found that individuals who experienced real threats showed higher aggressive intentions. Third, lower class groups are more likely to experience negative experiences and engage in aggressive behavior. According to the theory of social identity, after being classified into different social classes, lower-class individuals underwent more negative experiences related to their identities. Psychologically, lower-class individuals experience low self-esteem (Zhang and Zuo, 2006). To improve self-esteem, individuals may attempt to overthrow the original system through conflict behavior so that they have the same level of self-esteem as others (Jetten et al., 2017). Many studies have shown that individuals with low self-esteem show higher aggressive behavior (Ostrowsky, 2010; Garofalo et al., 2016; Amad et al., 2020). Therefore, we propose hypothesis 1: the competence stereotype held by the lower class toward the upper class positively predicts class conflict.

Considering the potential impact of competence stereotypes on class conflict, it is necessary to explore their mediating and moderating mechanisms. The BIAS Map theory proposes that the main prejudice caused by the competence stereotype is intergroup envy. Prejudice-related emotions play a mediating role between cognition and behavior (Cuddy et al., 2007). Therefore, the study of intergroup envy can help us understand its mechanism of action. In addition, the widening gap between the rich and the poor due to economic inequality is prone to class solidification (Peters and Jetten, 2023). And class solidification is one of the factors that predict class conflict (Zhang and Liu, 2017). Favorable class mobility can alleviate the negative impact of class solidification (Melita et al., 2023). Therefore, introducing the variable of belief in upward social mobility into the above mediation model, can help us understand the boundary conditions of class conflict induced by competence stereotypes. To sum up, the present study investigated intergroup envy as a possible mediator and upward social mobility belief as a possible moderator in the relationship between competence stereotypes and class conflict.

### 1.1 The mediating role of intergroup envy

Intergroup envy refers to anger and resentment caused by the fact that the in-group is not as well off as other groups in some respects (Cuddy et al., 2007; Smith and Kim, 2007). The cause of intergroup envy is not merely a desire to possess wealth, resources, or positive attributes but rather a perceived scarcity of these benefits in comparison to other groups (Jens et al., 2018). This emotion often appears in individuals in weak positions, and envious individuals tend to show more destructive behavior (Hamman, 2015). Relevant research has shown that when special circumstances cannot be improved, lower class people perceive a lack of control over their situation, and envy leads to more hostility (Rawls, 2009). Envy can also affect moral judgment (Polman and Ruttan, 2012). The envy generated when individuals face an unequal distribution leads to moral disengagement, that is, people develop cognitive rationalizations for engaging in immoral behavior (Hughes, 2007). This response is an important factor in destructive behavior. Studies have found that moral disengagement strongly mediates between envy and social undermining (Duffy et al., 2012). According to the relative deprivation theory, people feel deprived when they find themselves at a disadvantage by comparing their situation with a certain standard or reference. This sense of deprivation can produce negative emotions, also manifested as anger, resentment, or dissatisfaction (Mummendey et al., 1999). Studies have found that relative group deprivation significantly and positively predicts aggressive affect and behavior (Greitemeyer and Sagioglou, 2017). Other studies have shown that anger can lead to aggressive behavior (Berkowitz, 2012). Therefore, compared to the upper class, the lower class is at a disadvantage (Zhang and Liu, 2017), and lower class groups will feel a higher sense of deprivation or anger, which will increase the occurrence of conflict behavior (Berkowitz, 2012; Greitemeyer and Sagioglou, 2017).

In addition, the lower-class’s perception of higher-class competence may cause negative prejudice. According to the BIAS Map theory, prejudice caused by the competence stereotype mainly manifests as envy. The perception of competence stereotypes depends on income, wealth, education, work prestige, and the title of the group class (Fiske et al., 2002). In the context of economic inequality, people’s perceptions of group competence stereotypes have become stronger (Connor et al., 2021). As an emotional response to others’ wealth (Shamay-Tsoory et al., 2007), envy not only shows a strong desire for the wealth enjoyed by other groups, but also reflects the gap between one’s own and others’ status (Jankowski and Takahashi, 2014). The high-competence stereotype of the upper class symbolizes the wealth, status, and other objective resources possessed by this group. In the current era of unequal economic development, this is likely to trigger intergroup envy among the lower classes. Xia’s (2017) experimental study showed that lower class people have a higher tendency toward materialism. Individuals with higher materialism show higher envy (Żemojtel Piotrowska et al., 2013). Furthermore, according to the theory of social comparison, when people make upward social comparisons, they have reduced self-esteem when they are in a weak position in a certain respect (such as wealth or status), and then experience negative emotions such as pain and fear (Suls and Wheeler, 2012). Hu et al. (2023) found that self-esteem can negatively predict envy, implying that individuals with lower self-esteem tend to exhibit higher levels of envy. In summary, when the lower class compares themselves with the upper class in society, they often observe that the upper class possesses numerous desired possessions and privileges that they lack. This realization can further erode their self-esteem, ultimately triggering negative emotions (such as envy).

Furthermore, intergroup prejudice has been shown to play a mediating role in the relationship between intergroup stereotypes and behavioral tendencies. According to the social identity theory of economic inequality, with the aggravation of economic inequality, frequent wealth comparisons between groups will occur, which will enhance the perception of “us” and “them.” (Jetten et al., 2021). Therefore, differences between the upper and lower classes in terms of wealth are enhanced, resulting in higher competence stereotypes for the upper class (wealthy group) and lower competence evaluations for the lower class (Jetten et al., 2017). In today’s environment of fierce competition for resources, lower-class groups are more likely to have negative views of lower-class identity and experience lower self-esteem (Zhang and Zuo, 2006; Zuo et al., 2015). Individuals experience negative envy toward upper-class people to maintain their self-esteem. Hamman's (2015) study revealed that the anger sparked by negative jealousy can give rise to an urge to deprive or destroy “other people’s property.” In addition, a physiological study has shown that when the goal of envy is closely related to the individual’s self, it triggers the activation of brain-related regions, which represents the mechanism of conflict (Takahashi et al., 2009). Therefore, the stereotype of the competence of the upper class indicates this group’s objective survival resources and the competence to obtain them, which leads to envy in the lower class, thus increasing the likelihood of hostile behavior. Therefore, Hypothesis 2 was proposed: intergroup envy plays a mediating role in the positive prediction of competence stereotypes in class conflict.

### 1.2 The moderating role of upward social mobility belief

As mentioned previously, the competence stereotype of the upper class leads to an increase in class conflict through intergroup envy. Specifically, a series of emotions and behaviors generated by the lower-class stereotype of upper-class competence is due to the lack of objective material resources in some respects of the lower-class group, which, in turn, triggers the relevant psychological mechanism. In a socially stratified society, social status may change, which may be accompanied by psychological changes (Hochschild, 1996; Sagioglou et al., 2019). When individuals believe that social classes are permeable, they also believe their efforts can improve their social status (Moghaddam and Taylor, 1994; Melita et al., 2023). Specifically, an individual’s upward social mobility belief affects an individual’s current emotions and behaviors. Therefore, we examine whether the relationship between competence stereotypes and intergroup envy can be buffered by upward social mobility belief, thus reducing class conflict.

Belief in social mobility refers to an individual’s subjective perception of the degree of objective mobility (Kelley and Kelley, 2009). Compared with objective social mobility beliefs, subjective social mobility beliefs have a higher predictive power for individual behavior (Cheng et al., 2019). Social mobility beliefs are often divided into upward and downward mobility beliefs (Davidai and Gilovich, 2015). When upward mobility occurs, an individual’s subjective well-being can be improved by acquiring economic and social resources, such as individual income, opportunity, and social prestige (Bettina and Nadia, 2018; Zhang et al., 2019). Through an analysis of data from a British family group survey, Chan (2018) found that individuals who are also from the working class and have achieved upward mobility have higher scores on social support, subjective well-being, and other indicators than those who do not have mobility. Improved subjective well-being will cause individuals to experience fewer negative emotions (Tay and Diener, 2011; Yilmaz and Arslan, 2013), and produce more prosocial behavior (Kushlev et al., 2021). Therefore, we speculate that upward social mobility beliefs may reduce intergroup envy.

Upward social mobility beliefs can alleviate the influence of competence stereotypes on intergroup envy. Upward social mobility is an important way to advance individual interests and is an important manifestation of social equity (Zhang et al., 2019). Individuals’ upward social mobility is accompanied by the acquisition of economic and social resources such as income, opportunities, and social prestige (Zhang and Liu, 2017). This change enhances individuals’ sense of self-esteem and induces them to evaluate the upper class more positively. Higher self-esteem reduces envy (Hu et al., 2023). Thus, individuals with high upward social mobility beliefs are more likely to believe that society is equal. According to system justification theory, individuals with beliefs that justify the system, even if economic inequality in real society is high, may rationalize or even tolerate economic inequality. In particular, upward social mobility belief can improve people’s tolerance of economic inequality and life satisfaction (Cheung, 2016; Shariff et al., 2016), promote people’s political trust and general trust level (Cheng et al., 2012; Guo et al., 2019) and reduce envy. In a study of redistribution bias, Wakslak et al. (2007) experimentally manipulated people’s perception of class mobility and found that when people perceive higher class mobility, moral anger and negative emotions are significantly reduced. Therefore, we propose Hypothesis 3: in the model in which the competence stereotype of the upper class affects class conflict through intergroup envy, upward social mobility belief regulates the first half of the path. Specifically, for individuals with high upward social mobility beliefs, the level of intergroup envy predicted by high-level competence stereotypes is significantly reduced.

### 1.3 The present study

**Figure 1:**
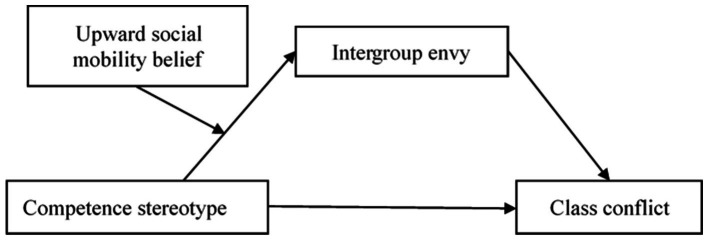
The proposed moderated mediation model.

Considering the adverse impact on the lower class of holding competence stereotypes of the upper class and its potential negative impact on class interaction in the context of economic inequality, it is necessary to examine the relationship between the competence stereotype of the upper class and class conflict and further explore its potential mechanism. In the present study, we examine lower-class students and focus on the mediating role of intergroup envy in the relationship between competence stereotypes and class conflict, and the moderating role of upward social mobility belief. Specifically, this study aimed to examine (a) whether competence stereotypes would positively correlate with class conflict, (b) whether intergroup envy mediates the relationship between competence stereotypes and class conflict, (c) whether upward social mobility belief moderates the relationship between competence stereotypes and intergroup envy, and (d) whether upward social mobility belief moderates the mediating effect of intergroup envy. In summary, we propose a moderated mediation model that can simultaneously explain the mediation (i.e., what is the relationship between competence stereotypes and class conflict?) and moderation mechanisms (i.e., when and for whom the connection becomes stronger or weaker). The proposed model is illustrated in Figure 1.

## 2 Materials and methods

### 2.1 Participants

The participants in this study were lower-class students from a university in Hubei, China. A total of 284, including 103 males (36.27%) and 181 females (63.73%). The mean age was 21.53 (*SD* = 1.73). According to G * Power 3.1 to estimate the expected effect size, 164 valid participants are required when the medium effect size is reached and the explanatory force is 99% (1 – *β* = 0.99, *α* = 0.01). The effective participants of this study meet the demanded quantity.

### 2.2 Procedure

Firstly, the research obtained approval from the scientific research ethics committee of our institution before data collection. From February 2023 to May 2023, a total of 1,623 questionnaires were distributed online (i.e., questionnaire stars and social networking sites) and offline (through campus recruitment). Before filling in the questionnaire, participants were provided with information that it was an anonymous survey on social attitudes and signed an informed consent form for this study. Additionally, participants were informed of their right to withdraw from the survey at any time without facing any consequences. Furthermore, we emphasized that the data collected in this study is solely intended for academic research purposes and will be treated with strict confidentiality. Participants were encouraged to respond honestly within a 25-min timeframe. Upon completion of the survey, participants received a small token of appreciation.

Secondly, we screened from 1,623 questionnaires, and 1,424 valid questionnaires. We eliminated invalid questionnaires based on the time participants took to complete the questionnaires and the quality of the questionnaires (not completed, choosing the same result for each question, answering carelessly). The valid recovery rate was 87.73%. Since our study was a low-class group, we performed a simple analysis of the objective economic status of these participants. Considering that students have no financial resources, we use the family economic level to measure (Johnson and Benson, 2012). According to the situation in China, there is a strong relationship between the objective economic status of class groups and occupation (Lu, 2002, 2004). We referred to the stratification technique used by many Chinese researchers (Hu et al., 2016; Ding et al., 2019). That is, individuals with an objective economic status score of less than 3 and below are low class, individuals with an objective economic status score of 4 to 6 are middle class, and individuals with an objective economic status score of 7 and above are high class. In 1424 valid data, we selected the low objective economic status of the participants 671 (47.12%). Because subjective economic status is more predictive of individual behavior (Hu et al., 2014), we re-screened participants with low objective economic status again. Finally, we got 284 absolutely low class participants (Subjective class score less than 3; Objective score less than 3: parents’ occupations are workers, farmers, or jobless individuals).

### 2.3 Measurements

#### 2.3.1 Objective economic status

The objective economic status measurement adopts the “Ten social classes” measurement developed by Chinese scholar Lu (2002). One event, ten points. This scale is the Chinese scholar Lu combined with China’s national conditions, according to occupational classification, organizational resources, economic resources, and cultural resources, Chinese society is divided into ten social classes (Lu, 2002, 2004). Each of the ten occupational categories represents a social class. For example, the unemployed, agricultural laborers, industrial workers, commercial service workers, individual businesses, government workers, professional technicians, private entrepreneurs, CEOs, and state administrators. Different occupations correspond to different scores ranging from 1 to 10. A higher score indicates a higher social class and vice versa.

#### 2.3.2 Subjective socioeconomic status

The Subjective Socioeconomic Status of the participants was measured by the classic subjective socioeconomic status MacArthur scale (Adler et al., 2000). One event, ten points. It is a ten-step ladder, each indicating the status of people with different levels of income, education, and professional prestige in the current society. In practice, first, participants were presented with a ten-level ladder diagram. Then told participants imagine the ladder represents the people in the social class status. The higher the rank, the higher the class status. Level 10 is the highest level of society. This group of people has the best living conditions, the highest income, the highest level of education, and the most decent jobs. Level 1 is the lowest level of society. This group has the worst living conditions, the lowest incomes, the least educated, and the most disreputable jobs. Finally, participants were asked to judge which rung of the ladder they were on based on their actual income, education level, and career status.

#### 2.3.3 Competence stereotypes

Competence stereotype is measured by the feature word rating method. The feature words about competence stereotypes come from Fiske et al. (2002) and Dai (2015), which have good reliability and validity. There were five feature words for the competence stereotype, namely “confident,” “capable,” “independent,” “smart,” and “efficient.” Participants rated 5 competence markers on a Likert scale, from 1(strongly disagree) to 7(strongly agree). All the scores are added together and averaged, and the higher the score, the more inclined the higher class is to coincide with these ability characteristic words. In this study, the Cronbach’s alpha coefficient is 0.888. Before filling in the score, according to the research on the typical high-class groups in China by various countries’ researchers (Zhou et al., 2015; Wu et al., 2018), we would tell the participants that the higher class groups were the second generation of officials, the second generation of rich people, coal bosses and so on.

#### 2.3.4 Intergroup envy

Intergroup envy was measured by Cuddy et al.'s (2007) scale of intergroup emotion. The scale consists of two dimensions of envy and jealousy (e.g., “From the point of view of the majority of the class, high class groups are envied.”; “From the point of view of the majority of the class, high class groups are jealousies.”). Participants respond to the 2 items on a Likert-type scale ranging from 1 (strongly disagree) to 6 (strongly agree). All scores are added together and averaged, higher scores represent a higher level of envy toward the higher class. The Cronbach’s alpha coefficient of the two items in this study was 0.684. Before filling in the score, we also would tell the participants that the higher class groups were the second generation of officials, the second generation of rich people, coal bosses, and so on.

#### 2.3.5 Upward social mobility belief

Upward social mobility belief measurement used the modified paradigm of Chinese scholar (Cheng et al., 2019), which was based on the study of Kraus and Tan (2015). The paradigm measured the individual’s upward social mobility beliefs by combining pictures and texts. First, participants were presented with class pictures (three classes, low, middle, and high) and told that there were currently 100 people in a class. Then let the participants evaluate the number of people who can successfully achieve three kinds of mobility (from the lower class to the middle class, from the lower class to the upper class, and from the middle class to the upper class) in every 100 people in Chinese society in the next decade. For example: “As the picture shows, there are currently 100 people in the lower class. How many people do you think will be able to move from the lower class to the middle class in ten years?.” A total of 3 items. When analyzing the data, convert it to a 10-point Likert scale. Then all scores are added together and averaged, higher scores represent a stronger upward social mobility belief. The Cronbach’s alpha coefficient of the three items in this study is 0.752.

#### 2.3.6 Class conflict

Class conflict is measured by Cuddy et al.’s (2007) scale of discriminatory behavior. This study used two dimensions of the scale, active harm and passive harm (e.g., “In everyday life, most people in my class would tend to attack higher-class groups.”; “In everyday life, most people in my class would tend to exclude higher-class groups.”). Participants respond to the 4 items on a Likert-type scale ranging from 1 (strongly disagree) to 6 (strongly agree). All scores are added together and averaged, higher scores represent more aggression toward the higher class. In this study, the Cronbach’s alpha coefficient of the four items was 0.845.

#### 2.3.7 Control variables

Gender and age were included as control variables in our model, as previous studies found that they were closely associated with the main variables in this study (Duehr and Bono, 2006; Ellemers, 2018).

### 2.4 Data analysis

In this study, a moderated mediation model was constructed with competence stereotypes as the independent variable, class conflict as the dependent variable, intergroup envy as the mediating variable, and upward social mobility belief as the moderating variable. Statistical analyses were conducted using SPSS 23.0. Due to the data of this study being self-reported by the participants, potential concerns regarding common method bias were addressed. Therefore, the Harman single-factor test was performed before the data analysis to test the potential common method bias (Podsakoff et al., 2003). The results showed that the eigenvalues of six factors were greater than 1. However, the first factor only explained 21.73% of the total variance and did not reach the critical standard of 40% (Zhou and Long, 2004), indicating that there was no common method bias in this study.

After common method bias evaluation, we carried out the following data processing steps. Firstly, we employed descriptive statistics and Pearson correlation analysis to examine the means, standard deviations, and bivariate associations of the study variables. Secondly, an independent sample t-test was adopted to examine the gender differences for the main variables. Thirdly, we tested the mediating effect by the Bootstrap method proposed by Hayes. Fourthly, the SPSS macro PROCESS (Model 7) Version 3.5 suggested by Hayes (2013) was used to test the moderated mediation model. This SPSS macro has been used to test mediating and moderating models in several studies, in which this SPSS macro showed higher statistical testability. Furthermore, simple slope analyses were performed to decompose all the potential significant interaction effects (Aiken and West, 1991).

## 3 Results

### 3.1 Preliminary analyses

Table 1 presents the means, standard deviations, and correlations for all of the observed variables.

**Table 1:** Descriptive statistics and interrelations among all of the observed variables.

Variables	*M*	*SD*	1	2	3	4	5	6
1. Gender	–	–	1					
2. Age	21.53	1.73	0.104	1				
3. Competence stereotypes	4.86	1.13	0.127^*^	0.125^*^	1			
4. Intergroup envy	4.22	1.08	−0.029	0.094	0.168^**^	1		
5. Class Conflict	3.62	1.05	−0.09	0.004	0.209^**^	0.430^**^	1	
6. Upward social mobility belief	2.54	1.87	0.112	0.005	0.063	−0.071	0.078	1

*N* = 284. ^**^p < 0.01, ^*^p < 0.05.

According to correlation analysis, competence stereotypes were positively correlated with both intergroup envy (*r* = 0.17, *p* < 0.01) and class conflict (*r* = 0.21, *p* < 0.01), but not with upward social mobility belief (*r* = 0.06, *p* > 0.05). Intergroup envy was positively correlated with class conflict (*r* = 0.43, *p* < 0.01) and not with upward social mobility belief (*r* = −0.07, *p* > 0.05). Upward social mobility belief was not correlated with class conflict (*r* = 0.08, *p* > 0.05). Furthermore, age was positively correlated with competence stereotypes (*r* = 0.13, *p* < 0.05), but not with other variables.

Besides, Table 2 presents the differences in the observed variables in gender. Results of the independent-sample *t*-test indicated that there were significant gender differences in competence stereotypes (*t* = −2.16, *p* < 0.05). This may be due to gender stereotypes, resulting in girls being more inclined to associate high class with masculine traits (ability, leadership, etc.) (Jaoul-Grammare, 2023). However, intergroup envy, class conflict, and upward social mobility belief all show no significant Gender differences.

**Table 2:** The differences of the observed variables in gender.

Variable	*M* ± *SD*	Competence stereotype	Intergroup envy	Class Conflict	Upward social mobility belief
Gender	Male	4.67 ± 1.32	4.26 ± 1.11	3.74 ± 1.17	2.27 ± 1.84
	Female	4.97 ± 0.99	4.20 ± 1.07	3.55 ± 0.96	2.70 ± 1.80
	*t*	−2.16*	0.49	1.51	−1.90

*p < 0.05.

### 3.2 Testing for the proposed moderated mediation model

**Figure 2:**
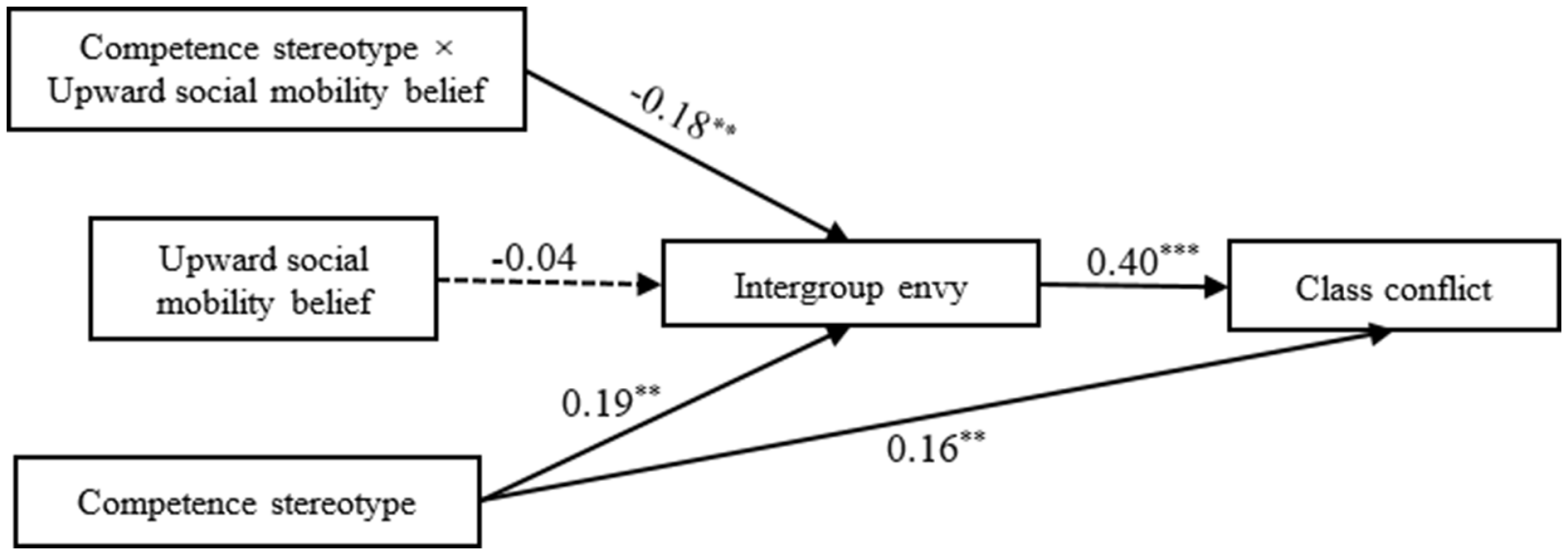
The relationship between competence stereotypes and class conflict: the mediating role of intergroup envy and the moderating role of perception of upward social mobility.

Hayes’s (2013) macro PROCESS was adopted to examine the proposed moderated mediation model. Figure 2 and Table 3 presented the main results of the moderated mediation analysis. As expected, the total effect model (*F* (4, 279) = 19.12, *R*^2^ = 0.22, *p* < 0.001), the mediator variable model (*F* (5, 278) = 4.77, *R*^2^ = 0.08, *p* < 0.001) were all significant after controlling gender and age.

**Table 3:** Regression results for the conditional indirect effects (moderated mediation).

Model											
*R*	*R^2^*	*F*	*df*_1_	*df*_2_	*p*	*β*	*SE*	*t*	*p*	LLCI	ULCI
*Model 1: total effect model (outcome variable: Class Conflict)*											
0.46	0.22	19.12^***^	4	279	<0.001						
Constant						0.81	0.68	1.20	>0.05	−0.53	2.15
Gender						−0.19	0.11	−1.74	>0.05	−0.41	0.03
Age						−0.02	0.03	−0.74	>0.05	−0.09	0.04
Competence stereotypes						0.16^**^	0.05	2.89	<0.01	0.05	0.27
Intergroup envy						0.40^***^	0.05	7.49	<0.001	0.30	0.51
*Model 2: Mediator variable model (outcome variable: Intergroup envy)*											
0.28	0.08	4.77^***^	5	278	<0.001						
Constant						−0.68	0.74	−0.93	>0.05	−2.13	0.77
Gender						−0.15	0.12	−1.25	>0.05	−0.39	0.09
Age						0.04	0.03	1.30	>0.05	−0.02	0.11
Competence stereotypes						0.19^**^	0.06	3.20	<0.01	0.07	0.30
Upward social mobility belief						−0.04	0.06	−0.63	>0.05	−0.15	0.08
Competence stereotypes × Upward social mobility belief						−0.18^**^	0.05	−3.33	<0.01	−0.28	−0.07
*The test of mediating effects*											
							Effect size	Boot *SE*	PME	Boot LLCI	Boot ULCI
Total effect							0.23	0.06	–	0.11	0.34
Direct effect							0.16	0.05	69.57%	0.05	0.27
The indirect effect of intergroup envy							0.07	0.04	30.43%	0.01	0.14
*Conditional indirect effect analysis at values of Upward social mobility belief (M ± SD)*											
								*β*	Boot *SE*	Boot LLCI	Boot ULCI
*M*-1*SD* (0.67)								0.15	0.06	0.04	0.26
*M* (2.54)								0.08	0.04	0.01	0.15
*M* + 1*SD* (4.41)								0.004	0.05	−0.09	0.10

*N* = 284. Bootstrap sample size = 5,000. *R*^2^, coefficient of determination; df, degree of freedom; β, unstandardized regression coefficients; SE, standard error; Cis, bootstrap confidence intervals; LLCI, lower limit of the 95% confidence interval; ULCI, upper limit of the 95% confidence interval; PME, Proportion of Mediating Effect ^**^p < 0.01,^***^p < 0.001.

Specifically, competence stereotypes positively predicted intergroup envy (*β* = 0.19, *SE* = 0.06, *p* < 0.01) and class conflict (*β* = 0.16, *SE* = 0.05, *p* < 0.01). Intergroup envy positively predicted class conflict (*β* = 0.40, *SE* = 0.05, *p* < 0.001). Furthermore, bootstrap 95% confidence intervals for the mediating effect of intergroup envy were found to not include 0, thus indicating that intergroup envy plays a significant mediating effect on completion stereotypes and class conflict, accounting for 30.43% of the total effect. These results provided compelling evidence that competence stereotypes were associated with an increase in class conflict and this relation was mediated by intergroup envy. Thus, Hypothesis 1 and 2 were supported.

To examine Hypothesis 3, the interaction effects were also analyzed with macro PROCESS (Model 7) by Hayes (2013). There was a significant competence stereotype × upward social mobility belief interaction effect on intergroup envy (*β* = −0.18, *SE* = 0.05, *p* < 0.01) in the mediator variable model. This finding indicated that the association between competence stereotypes and intergroup envy was moderated by upward social mobility belief.

**Figure 3:**
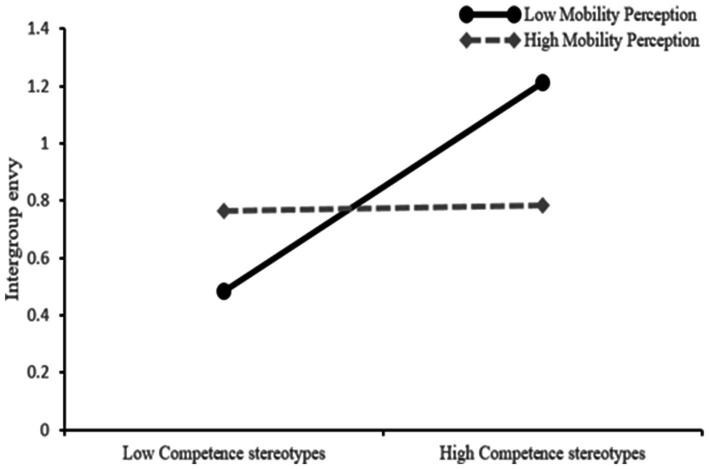
Mobility belief moderated the relationship between competence stereotypes and intergroup envy.

Additionally, simple slope analyses were conducted to illustrate this significant interaction and explore whether slopes for the high-mobility belief group (1 *SD* above the mean) were different from slopes for the belief group (1 *SD* below the mean) in the mediator variable model. The results are plotted in Figure 3. As shown in Figure 3, the effect of competence stereotypes on upward social mobility belief was positive and significant for college students with low mobility belief (*β* = 0.37, *t* = 4.41, *p* < 0.001), whereas it was not significant for those with high mobility belief (*β* = 0.01, *t* = 0.14, *p* > 0.05). The results indicated that the indirect effect of intergroup envy in the relationship between competence stereotypes and class conflict was stronger for individuals with lower mobility beliefs.

## 4 Discussion

In the era of deglobalization, unstable economic development leads to an increasing gap between rich and poor, and limited resource competition makes discrepancies between classes increasingly serious (Zhang and Liu, 2017). With growing awareness of class conflict, an increasing number of researchers are focusing on the potential risk factors for class conflict. As a cognitive factor, class stereotypes are significant predictors of conflict behavior (Hu et al., 2014), but the attention is limited. As a special group in the public makeup, the lower class plays an important role in the development of society (Havighurst, 1976; Bulmer, 2021). To fill these gaps, this study aimed to investigate the relationship between the stereotype of the upper class’s competence held by the lower class and class conflict and its potential mechanism. Specifically, we propose a moderated mediation model to analyze the role of intergroup envy and upward social mobility beliefs in the relationship between competence stereotypes and class conflict. Correlation analysis revealed that the competence stereotype held by lower-class s toward the upper class was positively correlated with class conflict. Thus, Hypothesis 1 was supported. In addition, the mediation analysis revealed the mediating role of intergroup envy between competence stereotypes and class conflict. Thus, Hypothesis 2 was supported. Moreover, a moderated mediation analysis showed that upward social mobility belief moderated the indirect effect of competence stereotypes on class conflict. In particular, lower-class with higher upward social mobility beliefs can successfully alleviate the negative impact of upper-class competence stereotypes on intergroup envy, thereby reducing class conflict. Thus, Hypothesis 3 was supported. The results of this study suggest that we can reduce the potential adverse effects of upper-class competence stereotypes on intergroup envy by enhancing upward social mobility beliefs, thereby reducing the risk of class conflict.

First, this study found that competence stereotypes held by the lower class toward the upper class positively predicted class conflict. BIAS map theory posits that people holding competence stereotypes of the upper class tend to cooperate and connect (Cuddy et al., 2007). However, this study found that competence stereotypes held by the lower class toward the upper class led to behavioral tendencies involving attack and injury. This may be related to the different social groups and competition situations. According to social identity theory (Zhang and Zuo, 2006; Hogg, 2016), in a general competitive situation, the group identity involvement of the lower class is low in intergroup interaction, and there is a certain relationship between the group’s warmth perception and aggressive behavior. However, in the context of special competition (e.g., economic inequality), the identity of the lower class is often activated, leading to a lower perception of warmth among the upper class. Moreover, the competence perception of the upper class is more predictive of attack behavior. Studies have found that in the context of economic inequality, people pay more attention to competence stereotypes (Connor et al., 2021). Fiske et al. (2002) found that Chinese people’s stereotypes of the upper class and high ability are highly correlated with discriminatory behavior. In addition, the lower-class group has more aggressive personality traits (Kraus et al., 2011). According to life history theory, long-term environmental shaping, including the strengthening of behavior, allows individuals to form a stable behavioral style (Guan and Zhou, 2016). From the perspective of material and psychological resources, the lower-class group has long been at a disadvantage, and thus adopts aggressive, confrontational, and risky adaptation strategies to meet current survival needs (Chang et al., 2019; Mengelkoch and Hill, 2020). Alcañiz-Colomer et al. (2023) proposed that lower income is predictive of higher levels of verbal or physical aggression. Griskevicius et al.’s (2011) study also found that lower class groups are more likely to adopt risky survival strategies than upper class groups. Therefore, in the context of unequal economic development, competence stereotypes held by the lower class of the upper class have a higher risk of predicting class conflict.

Second, consistent with previous studies showing that envy plays a mediating role in the relationship between stereotypes and aggressive behavior (Fiske et al., 2002; Cuddy et al., 2007), this study further shows from the intergroup perspective that intergroup envy can significantly mediate the relationship between competence stereotypes and class conflict. In other words, the competence stereotype held by the lower class toward the upper class will result in lower-class envy, which in turn will lead to class conflict. A considerable number of studies have supported the relationship between envy and aggressive behavior (Hamman, 2015) as well as the relationship between stereotypes and emotions (Cuddy et al., 2007). However, to the best of our knowledge, this is the only study to explore the mediating role of intergroup envy between competence stereotypes and class conflict behavior from a lower-class group perspective. At the same time, our results further verify that, from the lower-class group perspective, the competence stereotype of the upper class harms the individual’s psychology and behavior. In addition, this study showed that a positive stereotype held by the lower class of the upper class will produce negative emotions (such as envy), which will further lead to individual aggression (such as class conflict).

According to the stereotype content model, an individual’s perception of competence means their perception of economic status, social status, and power (Fiske et al., 2002). That is, the lower class’s perception of the higher competence of the upper class indicates that the latter group has a high social status while the former has a relatively low social status. According to the intergroup emotion theory of social identity, when individuals classify themselves as a lower-class group and accept this identity, they face fierce competition for resources in reality. This competition highlights the negative view of lower-class identity, which, in turn, causes individual self-esteem to decline. To maintain and improve self-esteem, individuals experience negative emotions (envy, anger, etc.) toward the upper class (out-group). This negative emotion prompts individuals to take measures to improve their self-esteem, such as aggressive behavior (Liu and Zuo, 2010). Amad et al. (2020) found that individuals with low self-esteem hurt others. From an evolutionary perspective, high competence, which means controlling more resources, can be regarded as an important tool for competing for limited resources (Fiske et al., 2002). Intergroup envy, an unconscious adaptive emotion in resource competition, contains hostile elements (Shamay-Tsoory et al., 2007). This will make individuals not care whether other people’s resource advantages are obtained fairly, and think that they deserve resources, while others should not obtain these resources. In other words, the lower class views the acquisition of resources as their entitlement, whereas they believe the upper class does not deserve their advantages and status. This belief generates hostility toward the upper class, motivating the lower class to attempt to eliminate their dominant position. The study found that when people experience an external threat to their survival, they will proactively address the crisis (Mobbs et al., 2015). In general, with the development of economic inequality, intergroup envy is a potential mechanism for understanding how the lower class’s stereotype of upper-class competence affects class conflict.

In addition, a more important finding of this study is that upward social mobility beliefs can moderate the relationship between the competence stereotype held by the lower class to the upper class and intergroup envy. Specifically, the competence stereotypes held by the lower class toward the upper class are directly moderated and buffered by upward social mobility through the indirect effect of intergroup envy on class conflict. Members of the lower class with robust upward social mobility belief exhibit a more pronounced version of this effect. This result may indicate that upward social mobility beliefs, as a positive cognitive factor, can help lower-class groups mitigate the negative consequences associated with positive stereotypes. With the alleviation of negative emotions, the risk of class conflict is reduced. Our results are consistent with those of previous studies indicating that social mobility perception is conducive to the harmonious development of society (Cheng et al., 2019). According to the social identity theory, social mobility, as a personal strategy, can change an individual’s social identity. Specifically, members of the lower class often disagree with their current status, leading to low self-esteem associated with their class identity. This, in turn, prompts a range of actions aimed at improving their status and achieving a target status that would enhance their self-esteem, thereby mitigating envy (Zhang and Zuo, 2006). Day and Fiske (2017) found that lower-class individuals with stronger beliefs in social mobility exhibit less willingness to engage in collective actions for social change. By manipulating social mobility beliefs, Sagioglou et al. (2019) found that lower-class individuals with high social mobility beliefs experienced lower hostility caused by relative deprivation, and reduced the generation of hostile acts (Berkowitz, 2012).

### 4.1 Limitations and implications

Although this study provides valuable findings for understanding how and when intergroup envy and class conflict are related to lower-class s’ stereotypes of upper-class competence, several limitations of this study need to be considered.

First, this study adopted a convenience sampling method and collected data only from college students at a domestic university. Therefore, the representativeness of the samples must be verified. Therefore, caution should be exercised when extending these findings to other populations. Future research could use random or stratified sampling methods to test the model using different social groups. Second, owing to the cross-sectional study design, causality can only be preliminarily established. Future research could use an experimental design to further test the causal relationships between competence stereotypes, intergroup envy, and class conflict. Third, this study only measured the individual’s level of upward social mobility belief and did not manipulate it to explore the role of upward social mobility belief. Considering the buffering and protective effects of upward social mobility beliefs on intergroup envy, it is necessary to conduct intervention research. Future research could enhance upward social mobility beliefs through experimental manipulations and examine the role of upward social mobility beliefs in the path by which competence stereotypes affect intergroup envy and class conflict. Finally, the data in this study were collected only through self-report questionnaires, which cannot avoid the influence of potential social desirability bias related to evaluations of competence and common method bias on the research results. Future research should use a multidimensional approach to collect more objective data.

Despite some limitations, this study holds considerable theoretical significance. Firstly, under the social background of the widening gap between the rich and the poor, this study explored that the reasons for the participation of the lower class in the class conflict are the positive stereotype of the higher class (competence dimension), the negative emotion of others’ wealth (intergroup envy). It found that the hope of “rebuilding” the social system—upward social mobility belief—can act as a buffer against these conflicts. Secondly, focusing on lower-class groups, this study integrated social identity theory with bias map theory, offering a theoretical contribution to explaining intergroup aggression among the lower classes. Within the framework of social identity theory, it further illuminated that class conflicts are directly driven by class stereotypes and prejudices. Meanwhile, the BIAS map theory allowed us to delve into the specific patterns of lower-class cognition, emotions, and behaviors. Thirdly, this study departed from the previous perspective of viewing class as a mere personality trait. Instead, based on intergroup interaction, this study regards class as an important group identity of individuals. In other words, whenever individuals perceive a negative group identity associated with the lower class, they are likely to develop corresponding intergroup cognitions, emotions, and behaviors. This approach significantly enriches the understanding of social class research from an intergroup interaction perspective.

In addition to these theoretical contributions, this study has several important practical implications. First, upward social mobility belief was an important buffer factor for individual intergroup envy. A higher level of upward social mobility belief attenuates the influence of upper-class competence stereotypes on intergroup envy so that it weakens or even disappears, resulting in a lower risk of class conflict. Therefore, guiding the lower-class groups to improve their upward social mobility beliefs deserves special attention. On one hand, lower-class individuals with low upward social mobility beliefs can be guided to pay attention to long-term goals through the media. This could help improve their perception of social mobility and stimulate the behavior of promoting upward mobility (Melita et al., 2023), to reduce the frequency or intensity of negative emotions experienced. On the other hand, relevant social departments can promote the implementation of social reform measures such as resource redistribution and facilitate more equitable opportunities for class mobility. Previous studies have found that promoting social equity and narrowing the gap between rich and poor are conducive to the harmonious development of society (Zhang and Liu, 2017). In summary, the negative effect on the lower class of holding competence stereotypes of the upper class can be buffered by improving an individual’s upward mobility perception.

Second, considering that intergroup envy plays a “bridging” role in the relationship between the competence stereotype of the upper class and class conflict, the risk of class conflict can be reduced by reducing intergroup envy among the lower class toward the upper class. In addition to changing emotions through the improvement of upward mobility perception, individuals can reduce their negative envy by improving self-efficacy. Previous studies have shown that improving self-efficacy can reduce an individual’s threat perception, thereby reducing envy (Ma, 2020). Self-efficacy can be improved by setting small goals and participating in physical exercise (Yuan et al., 2018), this effectively helps individuals experience positive emotions. Therefore, for lower-class individuals, forming good exercise or planning habits can be an effective strategy to improve intergroup envy and reduce class conflict.

## Data availability statement

The raw data supporting the conclusions of this article will be made available by the authors, without undue reservation.

## Ethics statement

The studies involving humans were approved by Ethics Committee of School of Psychology, Yangtze University, Jingzhou, China. The studies were conducted in accordance with the local legislation and institutional requirements. Written informed consent for participation in this study was provided by the participants’ legal guardians/next of kin. The animal studies were approved by Ethics Committee of School of Psychology, Yangtze University, Jingzhou, China. The studies were conducted in accordance with the local legislation and institutional requirements. Written informed consent was obtained from the owners for the participation of their animals in this study. Written informed consent was obtained from the individual(s), and minor(s)’ legal guardian/next of kin, for the publication of any potentially identifiable images or data included in this article.

## Author contributions

J-LL: Data curation, Formal analysis, Investigation, Writing – original draft. LY: Conceptualization, Funding acquisition, Methodology, Supervision, Writing – original draft, Writing – review & editing. Y-HZ: Funding acquisition, Supervision, Writing – review & editing. J-HG: Funding acquisition, Supervision, Writing – review & editing. L-CY: Supervision, Writing – review & editing.
